# Hepatitis C Virus Infection May Lead to Slower Emergence of *P. falciparum* in Blood

**DOI:** 10.1371/journal.pone.0016034

**Published:** 2011-01-10

**Authors:** Odile Ouwe-Missi-Oukem-Boyer, Fousseyni S. Touré Ndouo, Benjamin Ollomo, Jérome Mezui-Me-Ndong, Florian Noulin, Isabelle Lachard, Guy-Roger Ndong-Atome, Maria Makuwa, Pierre Roques, Michel Branger, Pierre-Marie Preux, Dominique Mazier, Sylvie Bisser

**Affiliations:** 1 Centre International de Recherches Médicales de Franceville, Franceville, Gabon; 2 Hôpital Bichat-Claude-Bernard, Paris, France; 3 Université de Limoges, IFR 145 GEIST, Institut de Neurologie Tropicale, EA 3174 NeuroEpidémiologie Tropicale et Comparée, Limoges, France; 4 INSERM, U945, Paris, France; 5 Université Pierre et Marie Curie-Paris6, UMR S945, Paris, France; 6 AP-HP, Groupe hospitalier Pitié-Salpêtrière, Service Parasitologie-Mycologie, Paris, France; 7 Division of ImmunoVirology (SIV), Institute of Emerging Diseases and Innovative Therapies (IMETI), CEA, Fontenay-aux-Roses, France; 8 UMR E1, University Paris Sud XI, Orsay, France; Federal University of São Paulo, Brazil

## Abstract

**Background:**

Areas endemic for *Plasmodium falciparum*, hepatitis B virus (HBV) and hepatitis C virus (HCV) overlap in many parts of sub-Saharan Africa. HBV and HCV infections develop in the liver, where takes place the first development stage of *P. falciparum* before its further spread in blood. The complex mechanisms involved in the development of hepatitis may potentially influence the development of the liver stage of malaria parasites. Understanding the molecular mechanisms of these interactions could provide new pathophysiological insights for treatment strategies in Malaria.

**Methodology:**

We studied a cohort of 319 individuals living in a village where the three infections are prevalent. The patients were initially given a curative antimalarial treatment and were then monitored for the emergence of asexual *P. falciparum* forms in blood, fortnightly for one year, by microscopy and polymerase chain reaction.

**Principal Findings:**

At inclusion, 65 (20.4%) subjects had detectable malaria parasites in blood, 36 (11.3%) were HBV chronic carriers, and 61 (18.9%) were HCV chronic carriers. During follow-up, asexual *P. falciparum* forms were detected in the blood of 203 patients. The median time to *P. falciparum* emergence in blood was respectively 140 and 120 days in HBV- and HBV+ individuals, and 135 and 224 days in HCV- and HCV+ individuals. HCV carriage was associated with delayed emergence of asexual *P. falciparum* forms in blood relative to patients without HCV infection.

**Conclusions:**

This pilot study represents first tentative evidence of a potential epidemiological interaction between HBV, HCV and *P. falciparum* infections. Age is an important confounding factor in this setting however multivariate analysis points to an interaction between *P. falciparum* and HCV at the hepatic level with a slower emergence of *P. falciparum* in HCV chronic carriers. More in depth analysis are necessary to unravel the basis of hepatic interactions between these two pathogens, which could help in identifying new therapeutic approaches against malaria.

## Introduction

Areas where malaria, hepatitis B (HBV) and hepatitis C (HCV) are prevalent frequently overlap in sub-Saharan Africa [1], [2], [3]. However, studies of *Plasmodium* and hepatitis virus coinfection are rare [4], [5], [6], [7] despite the fact that liver-stage malaria parasites, HBV and HCV viruses all develop in hepatocytes and may thus interact. The simultaneous presence of both pathogens in the liver could lead to two “opposing” phenomena: reduced HLA class I expression on infected hepatocytes that would protect the developing hepatic malaria parasite from destruction [8], or inhibition of the development of the hepatic malaria parasite through the induction of non-specific inflammatory factors. The fact that different molecules such as CD81 [9], Scavenger Receptor B1 [10] are implicated in some of the early invasive steps of hepatocytes by hepatitis virus and *P. falciparum*, further makes functional investigation of co-infected hosts highly interesting.

We therefore conducted a prospective longitudinal study designed to determine whether HBV or HCV infection interferes with pre-erythrocytic development of *P. falciparum*. This was done by assessing the delay of emergence of malaria parasites in the blood after a radical cure, such a delay denoting an alteration in hepatocyte receptivity to the parasite [11], [12]. We studied individuals living in Dienga, a village located in an area of south-eastern Gabon where malaria is hyperendemic and where preliminary studies have shown high HBV and HCV seroprevalence rates ([13] and Makuwa et al, unpublished data).

## Methods

### Study area

The study took place from February 2003 to March 2004 in Dienga, a village located in Ogooué Lolo province (south-eastern Gabon, near the Congo border), approximately 200 km (a 3-hour drive) from Franceville. The population of Dienga belongs to the Banzabi ethnic group [14]. In 1994 a fully equipped field base was established in this village of nearly 2500 inhabitants, and three permanent CIRMF team members ensure full-time monitoring of study cohorts [15]. Malaria is highly endemic in this rain forest area, with seasonal peaks of transmission coinciding with the rains, from February to June and from September to December. The main malaria species is *P. falciparum* (>80%) and transmission is predominantly due to *Anopheles gambiae*, with an estimated 100 infective bites per person per year [16].

### Patients

Individuals aged 13–85 years residing permanently in Dienga were the focus of this study. All volunteers were interviewed in French or the local language and received a physical examination. Written informed consent was obtained from each volunteer or tutor before enrolment. The exclusion criteria were i) known severe illness other than malaria or hepatitis, ii) first quarter of pregnancy, iii) likely problems with follow-up; and iv) known hypersensitivity to sulfadoxine-pyrimethamine (SP) and/or artesunate (AS). A total of 319 individuals were enrolled, and each was attributed a unique number for this study.

### Study design

Baseline (day 0) blood samples were collected from each volunteer, and 2 to 4 aliquots of serum, ethylenediaminetetraacetic acid (EDTA)-plasma and buffy coat were frozen at −20°C and/or in liquid nitrogen for transportation to CIRMF headquarters for biochemical, parasitological and virological analyses. A thick blood smear was prepared for direct examination and three drops of blood were deposited on calibrated pre-punched paper disks (Serobuvard, LDA^22^®, Zoopole, Ploufragan, France) [17] for molecular studies. After 4 h at room temperature, the dried blood spots (DBS) were placed in envelopes, transported to CIRMF, and stored in an air-conditioned room until use. In keeping with WHO recommendations [18], all the volunteers were given oral artemisinin-based combination therapy (ACT) as follows: one dose of 25 mg/kg sulfadoxine plus 1.25 mg/kg pyrimethamine on day 0, and three doses of AS 4 mg/kg (Arsumax®, Sanofi, France), on days 0, 1 and 2 [19]. All the subjects were directly observed for at least 30 min following the intakes and were given a new dose if vomiting occurred.

Each volunteer was given an individual appointment calendar mentioning the date of inclusion and the dates of planned fortnightly follow-up visits to the CIRMF field base. Volunteers who missed an appointment were visited at home by a team member to prepare a blood smear and DBS. Volunteers who were absent were actively sought in order to avoid missing data. Each volunteer was given an individual card summarizing his or her personal information and all parasitological results. Patients found to be parasitemic either during the follow-up period or on any day after initial treatment were interviewed and physically examined before being treated with SP if asymptomatic, or with quinine (Quinimax® tablets for seven days) if symptomatic (fever ≥37.5°C and parasitemia >5000 p/µl of blood and no apparent other cause of fever [20]). Patients were excluded from the study once they had been treated. The entire population of Dienga was invited to see a doctor at the CIRMF base at any time during the study period and, when necessary, received medications and/or laboratory tests free of charge. Moreover, in 2005, HBV vaccination was proposed for every child under 5 years of age in Dienga (about 200 children were vaccinated). Individuals with clinical or biological signs of hepatitis were followed closely. The project was approved by the Gabonese Ministry of Public Health, the governor of the province, the local prefect, and the village authorities. Written informed consent was obtained from all participants prior enrolment. All clinical investigations have been conducted according to the international ethical standards.

### Laboratory testing

#### Diagnosis of HBV and HCV infections and transaminase assays

They were performed at CIRMF, on samples taken on day 0. Plasma samples were used for HBV and HCV testing. Anti-HCV antibodies were detected with a third-generation enzyme immunoassay (Monolisa anti-HCV Plus version 2, Bio-Rad, Marnes-La-Coquette, France). Positive and doubtful samples were retested with a third-generation recombinant strip immunoblot assay (RIBA, Chiron Corporation, Emeryville, CA). Samples positive in both tests were tested for HCV RNA by using qualitative reverse transcriptase polymerase chain reaction (PCR) amplification of the 5′ non coding region, as previously described [13], [21], [22]. All HCV RNA-positive individuals were considered to be HCV chronic carriers. Total anti-HBc antibodies and HBsAg were detected with enzyme immunoassays (Monolisa anti-HBc Plus and Monolisa Ag HBs Plus, Bio-Rad, Marnes-La-Coquette, France). Subjects positive in both tests were considered to be HBV chronic carriers.

Serum concentrations of glutamic oxalacetic and glutamic pyruvic transaminase (SGOT and SGPT) were determined with a Hitachi 902 analyzer (Roche Diagnostics, Mannheim, Germany) and aminotransferase activities were determined with a kinetic method (Enzyline GOT and Enzyline GPT respectively, Biomerieux, Marcy l'Etoile, France). SGOT and SGPT levels were considered abnormal if above 40 IU/L.

#### Malaria

It was diagnosed by microscopic examination of thick blood smears stained with Giemsa, and by PCR.

Microscopic examinations were performed at CIRMF by three pairs of readers, each consisting of one senior microscopist and one laboratory technician. The pairs of readers were alternated on a weekly basis, following a preset schedule. Positive smears were examined against 200 white blood cells, and parasite density was calculated as the number of parasites per microliter of blood, based on a putative mean white blood cell count of 8000/µL. Samples were only considered negative after examining the entire thick smear. A single *P. falciparum* parasite was detected in some samples, giving an estimated limit of detectable parasite density of 0.1 p/µL, assuming that about 10 µL of blood was used to prepare a thick smear. Each positive slide was systematically confirmed by all three senior microscopists. Routine quality controls were implemented, whereby 10% of randomly selected smears were re-examined, blindly to the initial result, by one of the three senior microscopists, on a rotating schedule (never the senior microscopist who initially examined the slide). Individuals with positive blood smears during follow-up were treated and subsequently excluded from the study.

Microscopy-negative samples were submitted to PCR targeting the gene encoding *P. falciparum* SSUrRNA, as previously described [23]. PCR could not distinguish between asexual and sexual parasites.

#### DNA extraction

Template DNA was extracted from DBS by using Chelex100, as described by Plowe [24].

#### DNA amplification

Each DNA sample (2.5 µL) was amplified in a Perkin Elmer thermal cycler. Amplification was carried out in a final volume of 25 µL containing 1xPCR buffer (as supplied by the manufacturer: 200 mM Tris, 100 mM KCl, 100 mM (NH_4_)_2_SO_4_, 20 mM MgSO_4_, 1% Triton x100, 1 mg/mL bovine serum albumin), 200 µM each dNTP (dATP, dCTP, dGTP and dTTP), 1 µM of each primer and 1 unit of Taq DNA polymerase (Invitrogen, Cergy Pontoise, France). First- and second-round amplifications were performed as reported by Snounou et al [23]. After amplification, 15 µL of each nested PCR product was analysed by electrophoresis on 1.5% agarose gel and visualized by UV transillumination after staining with GelRed (Interchim, Montluçon, France). A specific DNA band of 205 base pairs signals *P. falciparum* infection.

#### Assessment of potential antimalarial self-medication

The patients were asked not to use antimalarial drugs during the follow-up period. To evaluate possible self-medication, the patients were individually interviewed using a questionnaire. The use of antimalarial drugs was also evaluated with the Saker-Solomons test for 4-aminoquinoleine metabolites in urine samples from 53 randomly chosen subjects [25].

#### Assessment of the time to *P. falciparum* emergence in blood

The time to *P. falciparum* emergence was defined as the period (in days) between the end of the initial potentially curative treatment (on D0) and the detection of *P. falciparum* blood stages, irrespective of clinical status. Blood stages of *P. falciparum* were considered to have emerged at the midpoint between the last negative and the first positive diagnostic test (microscopy or PCR) after initial curative treatment. Follow-up was terminated if patients had at least two/three or more consecutive missing data points (corresponding to an absence of at least one month) and if antimalarial self-medication was detected by interview or metabolite assay. These patients were considered as being uninfected until the day before follow-up was terminated.

### Statistical analysis

All data were entered and verified with Excel or Access software (Microsoft). Statistical analyses were performed with Epi-Info 6.0 (CDC, Atlanta, GA) and Statview 5.0 (SAS Institute Inc., Cary, NC). Descriptive analysis used means ± standard deviation (SD) for quantitative variables and proportions (%) for qualitative variables. Means were compared using Student's *t* test and proportions were compared using the chi-square test. The chi-square test for trends was used to estimate prevalence trends across the different age groups. Survival rates were estimated with the Kaplan-Meier method and compared using the log-rank test, according to sex, age and hepatitis virus serostatus. Cox multivariate analysis included all variables with univariate *P* values of <0.25, and a stepwise backwards procedure was used until the final model was obtained. The level of significance was set at 5% for all analyses.

## Results

A total of 319 individuals (136 males and 183 females, sex ratio 0.74) aged from 13 to 85 years (mean ± SD  = 38.2±18.6 years) were included in this study (Table 1). The predominance of females (57.4% versus 42.6%; *P* = 0.0004) reflected the sex distribution of the entire population of Dienga at the time of the study (data not shown).

**Table 1:** Characteristics of the whole cohort and of participants according to HBV and HCV serostatus.

Characteristics	Whole cohort	HBV chronic carriers	HBV neg	HVC chronic carriers	HCV neg
n (%)	319 (100)	36 (11.3)	283 (88.7)	61 (19.1)	258 (80.9)
male (%)	136 (42.6)*	22 (61.1)	114 (40.3)	24 (39.3)	112 (43.4)
female (%)	183 (57.4)	14 (39.9)	169 (59.7)	37 (61.7)	146 (56.4)
age range	13–85	15–57	13–85	13–85	15–78
mean age (+/−SD)	38.2 (+/−18.6)	27.0 (+/−9.9)	39.6 (+/−18.9)	57.7 (+/−14.3)	33.6 (+/−16.3)
range GOT	6–609	7–249	6–609	9–154	6–609
mean GOT (+/−SD)	26 (+/−40)	36 (+/−43)	25 (+/−40)	31(+/−25)	25 (+/−43)
range GPT	5–1473	8–289	5–1473	8–163	5–1473
mean GPT (+/−SD)	25 (+/−84)	31 (+/−50)	25 (+/−88)	26 (+/−23)	25 (+/−93)
abnormal levels of transaminases n (%)	32 (10.1)	7 (19.5)	25 (8.9)	13 (21.7)	19 (7.4)

*P* = 0.0004

£ two persons not tested.

### Hepatitis virus serostatus (Table 1)

Of the 319 subjects, 36 (11.3%; age 15–57 years, sex ratio 1.57) were HBV chronic carriers and 61 (19.1%; age 13–85 years, sex ratio 0.65) were HCV chronic carriers. Two women (36 and 43 years) carried both viruses. These two patients were included in both the HBV and HCV carrier groups and were not analyzed as a distinct group. Transaminase levels were elevated in 32 individuals (10.1%), of whom seven were HBV carriers (19.5% of this group) and 13 were HCV carriers (21.7% of this group). The remaining 12 patients were HBV- and HCV-negative.

### Malaria status (Figure 1)

**Figure 1:**
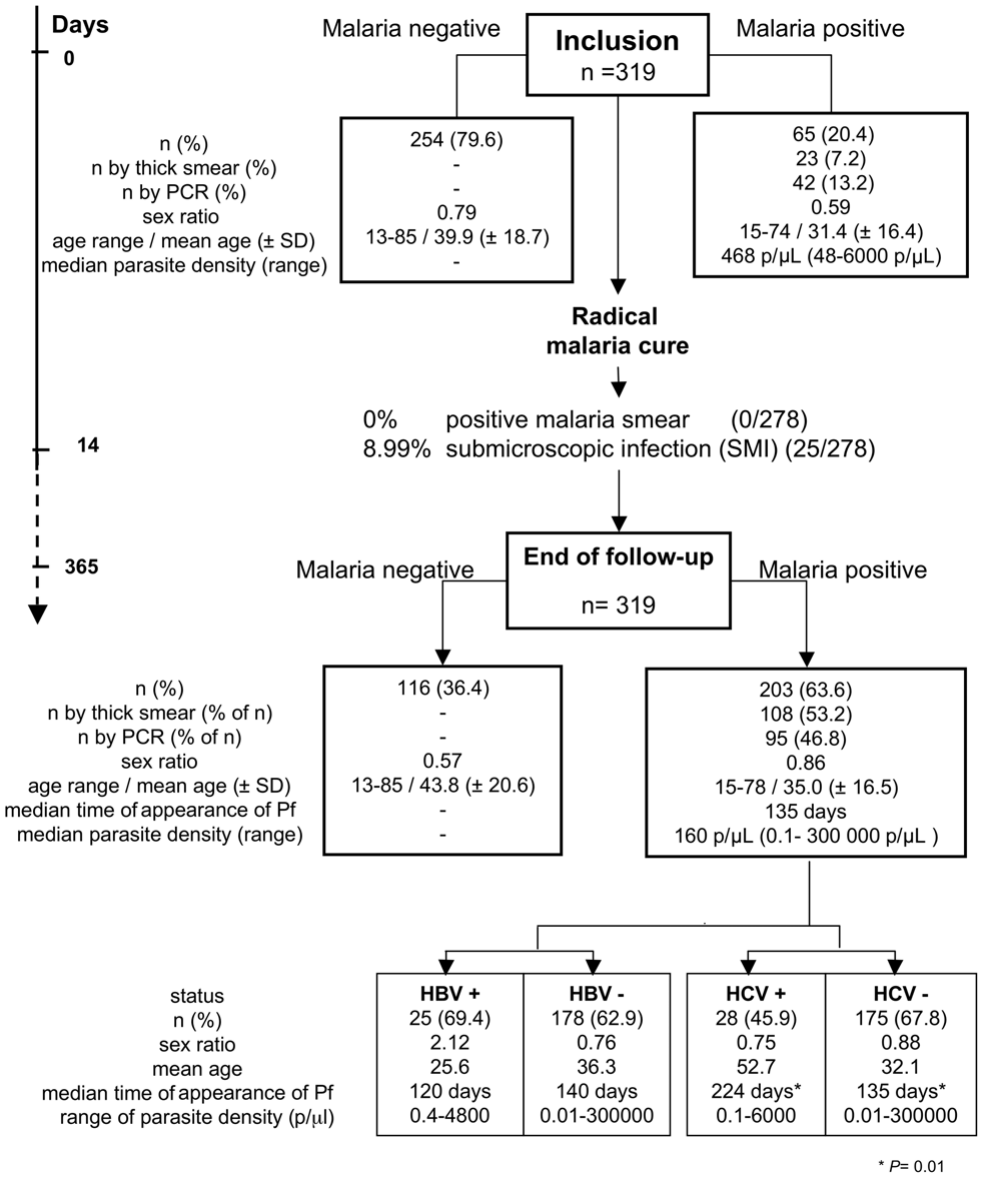
Schematic representation of the study. The flow chart represents the total number of patients enrolled, and analyzed, and the status of all patients at one-year follow-up.

Sixty-five cases of *P. falciparum* infection (20.4% of the cohort) were diagnosed before treatment, 23 (7.2%) by microscopy and 42 (13.2%) by PCR (Figure 1).

Two weeks after initial treatment, 278 (87.14%) patients were examined and all had negative thick blood smears, while 25 patients (8.99%) had submicroscopic infection (SMI) [26]. Among these 25 PCR-positive, smear-negative patients, three had genetically identical *P. falciparum* strains before and after treatment and thus were considered to have treatment failure.

During the one-year follow-up period, 203 cases of *P. falciparum* infection (63.6%) were diagnosed, 108 by microscopy and 95 by PCR (Figure 1).

**Figure 2:**
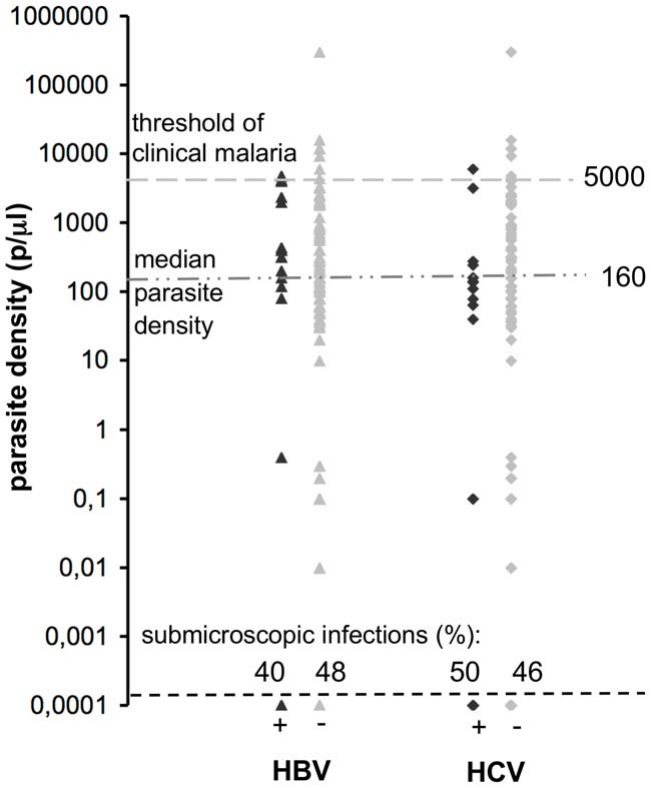
Parasite density distribution according to hepatitis virus serostatus. Parasite density distribution in the 203 patients in whom *P. falciparum* blood forms emerged after the last day of initial curative treatment, according to HBV serostatus (▴, 25 positive; 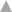, 178 negative) and HCV serostatus (♦, 28 positive; 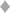, 175 negative). The median parasite density in the 203 subjects (160 p/µL) is indicated by a horizontal dashed line. The dotted line at 5000 p/µL corresponds to the threshold of clinical malaria.

Among the 203 subjects who were positive by direct examination or PCR after initial treatment, 25 were HBV carriers (69.4% of the 36 HBV carriers). Parasite density ranged from 0.4 to 4800 p/µL and all these subjects were asymptomatic (Figure 2). Twenty-eight of the 203 patients were HCV carriers (45.9% of the 61 HCV carriers). Parasite density ranged from 0.1 to 6000 p/µL and one patient was symptomatic (Figure 2).

Self-treatment was detected by 4-aminoquinoline metabolite assay in 11.3% of the tested individuals (data not shown).

### Age distribution (Figure 3)

Most HBV carriers were younger than 30 years and only one was older than 45 years. The HBV-Ag seroprevalence rate fell markedly with age (*P*<0.0001) and HBV carriers were significantly younger than HBV-Ag negative subjects (27.0±9.9 versus 39.6±18.9 y; *P* = 0.0001). By contrast, the HCV seroprevalence rate increased markedly with age (*P*<10^−5^) and HCV carriers were significantly older than HCV-negative subjects (57.7±14.3 versus 33.6±16.3 years; *P*<10^−5^).

**Figure 3:**
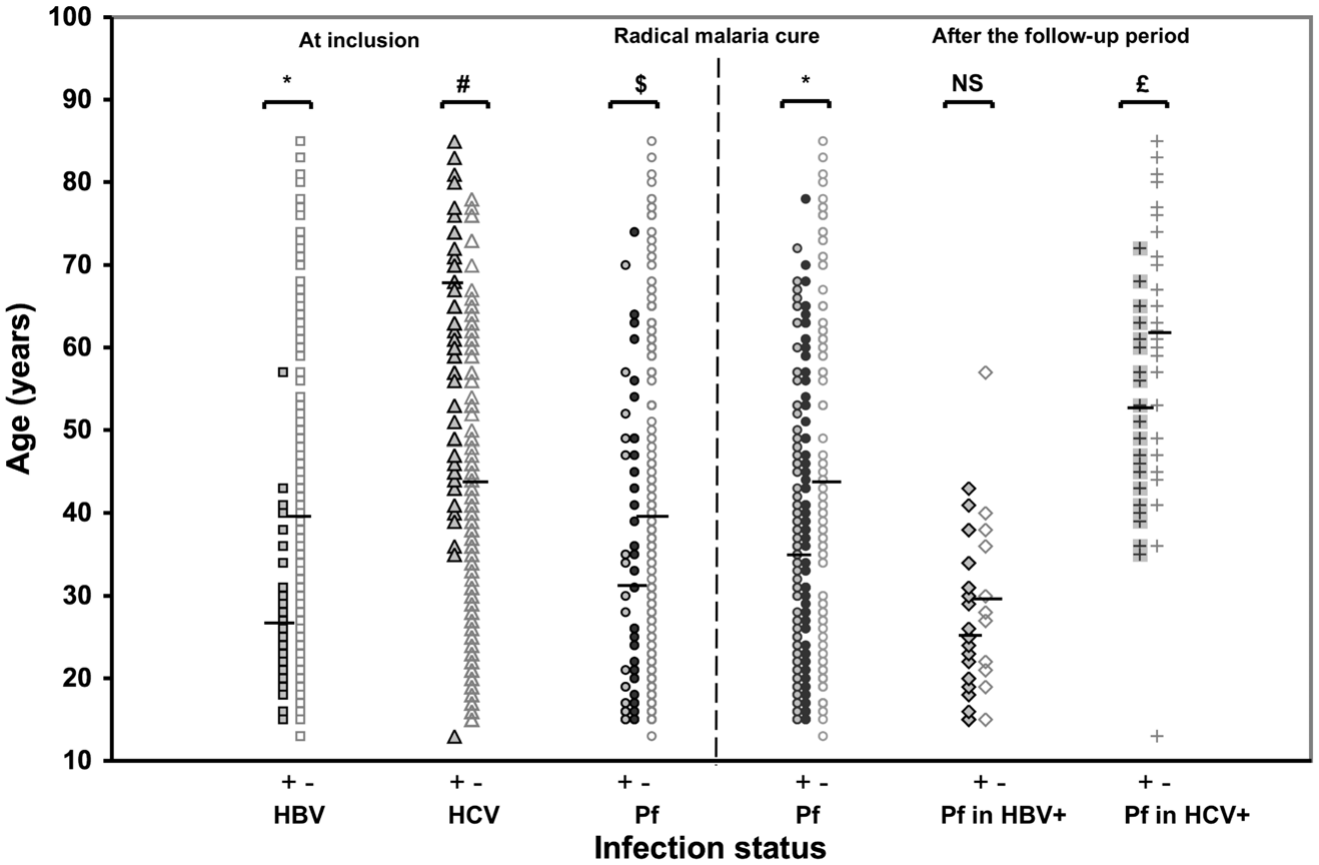
Age distribution according to infection status. * *P*≤0.0001. # *P*<10^−5^. $ *P* = 0.001. £ *P*<0.002. NS, not significant.

The 65 subjects with microscopic or submicroscopic *P. falciparum* infection before treatment were significantly younger than the other subjects (31.4±16.4 years versus 39.9±18.7 years; *P* = 0.001). Similarly, the 203 subjects with post-treatment *P. falciparum* infection were significantly younger than the other subjects (35.0±16.5 versus 43.8±20.6 years; *P*<0.0001). There was no significant age difference between the subjects diagnosed with *P. falciparum* infection before initial treatment and during post-treatment follow-up (31.4±16.4 versus 35.0±16.5 years; *P* = 0.35).

The 25 *P. falciparum*/HBV-coinfected subjects were younger than the other 11 HBV-infected subjects, but the difference was not statistically significant (25.6±8.8 versus 30.3±12.0 years; *P*<0.2). In contrast, the 28 *P. falciparum*/HCV-coinfected subjects were significantly younger than the other 33 HCV-infected subjects (52.7±11.3 versus 62.0±15.4 years; *P* = 0.002).

### Time to *P. falciparum* emergence

**Figure 4:**
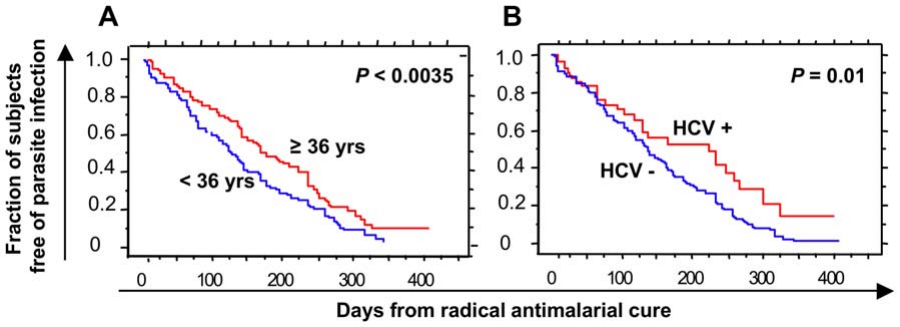
Kaplan-Meier Survival Curves for the Patients According to (A) Age or (B) Hepatitis C Virus serostatus. (A) in blue fraction of patients ≤36 years old free of infection, in red patients ≥36 years old free of infection. (B) in blue fraction of patient HCV negatives free of infection, in red fraction of patient HCV positive free of infection.

Blood forms of *P. falciparum* emerged a median of 135 days after initial treatment. This time did not differ by gender (univariate analysis, *P* = 0.2; data not shown) but was significantly related to age: subjects under 36 years (the median age) were infected more rapidly than older subjects (*P*<0.0035; Fig. 4A).

The median time to *P. falciparum* emergence was shorter among HBV-positive patients (120 d) than among HBV-negative patients (140 d), although the difference was not statistically significant (*P* = 0.1). In contrast, the median time to *P. falciparum* emergence was significantly longer (224 d) in HCV-positive patients than in HCV-negative patients (135 d; *P* = 0.01) (Figure 4B). The median time to parasite emergence was also significantly longer among subjects with stated or proven self-medication than among other subjects (247 versus 117 days; *P*<0.0001).

### Multivariate analysis

HCV status, age and self-medication were significantly associated with the time to *P. falciparum* emergence in univariate analysis and were therefore included in the Cox proportional hazards model. *P. falciparum* emergence was mainly retarded by self-medication (*P*<0.0001) and, to a lesser extent, by HCV coinfection (*P* = 0.0669), while age was clearly identified as a confounding factor (Table 2).

**Table 2:** Cox proportional hazard model.

COX MODEL	HR	CI 95%	P
Self-medication	0,44	(0,29–0,63)	<0,0001
HCV co-infection	0,68	(0,46–1,02)	0,0669

Legend: HR: hazard ratio; CI: confidence interval; P: P value.

## Discussion

This study was designed to provide first epidemiological evidence of a possible interaction at the hepatic level between *P. falciparum* and hepatitis viruses. The success of this study was largely due to the fact that the population of Dienga has been involved in medical studies for more than 20 years and participated actively.

The prevalence of HBV infection was 11.3%, but it should be noted that children under 13 years of age, who usually have the highest prevalence, were not included [27]. The HBV-Ag seroprevalence fell markedly with age, in keeping with the results of pioneering surveys of several rural areas of Gabon [28], [29]. The HCV seroprevalence was 18.9%, confirming the strong HCV endemicity reported in this equatorial region [29], [30], [31]. The HCV seroprevalence increased gradually with age, in accordance with the markedly different age distribution of HCV and HBV infection [30]. This different age distribution may explain the low prevalence of HBV/HCV coinfection in our study (only two cases).

During the one-year follow-up period after curative antimalarial treatment, 203 cases of *P. falciparum* infection were diagnosed, representing 63.6% of the cohort. Among these, 46.8% of infected individuals were SMI detected by PCR, indicating that microscopic examination is a poor indicator of *P. falciparum* infection [32], [33], [34]. However, the detection of SMI may have been underestimated since the PCR targeting the stevor gene has been recently reported to be more sensitive than the one using the SSUrRNA gene [35]. Parasitological surveillance tests were performed fortnightly in this large study (319 subjects), rather than weekly in previous studies investigating the re-emergence of *P. falciparum* after radical antimalarial therapy, but these studies usually involved shorter follow-up and/or smaller cohorts [36], [37], [38], [39]. The median time to *P. falciparum* emergence was 135 days in our study, compared to 115 days in a study of Dienga schoolchildren [36] but it should be noted that children are more susceptible to infection than adults [40].

HCV carrier status but not HBV carrier status was significantly associated with slower emergence of malaria parasites in univariate analysis, along with older age and self-medication. The relation with HCV carrier status was confirmed in multivariate analysis, although the *P* value was just above the threshold of significance. As age is a confounding factor, the influence of HCV infection on the natural course of *P. falciparum* malaria would be best examined in a case-control study, as in a previous study of HBV coinfection in the Gambia [41]. In this case-control study, Thursz et al. showed that HBV virus carriage was significantly increased amongst cases of severe malaria compared to matched controls. Barcus et al.[4] found similar results in Vietnamese patients. It was further postulated that this could be due to reduced HLA class I expression at the surface of hepatocytes that would then lead to a reduced destruction of intra-hepatic parasites by MHC-restricted cytotoxic mechanisms, which are known to play an important role in the control of malaria liver stages. Without discarding any subversion of the immune system by HCV [8], we can propose that the “protective” effect of HCV carriage may also be a consequence of non-specific mechanisms/mediators such as MHC-independent cytotoxic mechanisms [42], [43]. Furthermore, a number of non-specific inflammatory factors are known to be capable of inhibiting the development of *Plasmodium* in the hepatocyte: cytokines, reactive oxygen or NO derivatives, acute phase proteins in general (reviewed in [44], [45]).

All the above non-specific factors are observed during a viral infection [46], as the infected cells secrete cytokines and chemokines that lead to the recruitment and activation of inflammatory cells to the infected tissue. This is the case for NK cells, and possibly IFN-γ-secreting TNK lymphocytes. Subsequently, lymphocyte populations also migrate to the liver, and in addition to their cytotoxic activity directed against infected cell, they release IFN-γ and TNF-α. The role of these two cytokines is critical for the recruitment and activation of macrophages, NK cells and T lymphocytes, which can in turn produce cytokines and monokines. This inhibitory effect of the non-specific effectors on intra-hepatic *Plasmodium* parasites is not surprising, such “cross-pathogen” defense mechanisms have been described in transgenic mouse models developed by the group of Chisari [5], [47]. Finally, it has been shown that different host molecules such as the tetraspanin CD81 [9] and the Scavenger Receptor B1 [10], were not only implicated in certain key steps of the internalization of the virus but also of *P. falciparum* in hepatocytes as well. It would be indeed astonishing if there were no interactions at play during a combined *Plasmodium*/hepatitis infection. Differences in HBV and HCV chronic infections need to be further studied to understand the specific mechanisms in play. *In vitro* studies are underway to unravel the cellular and molecular basis of this interaction between HCV and *P. falciparum*.
